# Video review of family medicine resident clinical encounters: a tool for building emotional intelligence

**DOI:** 10.3389/fpsyg.2023.1188041

**Published:** 2023-07-11

**Authors:** Aubry N. Koehler, Mark P. Knudson, Parissa J. Ballard, Linda M. Nicolotti, Elimarie Caballero-Quinones, Stephanie S. Daniel

**Affiliations:** School of Medicine, Wake Forest University, Winston-Salem, NC, United States

**Keywords:** family medicine residents, emotional intelligence, video review, residency education, patient centered care

## Abstract

Video Review (VR) is a well established educational tool for developing the practice of patient-centered care in family medicine residents. There are a number of behaviorally-based checklists that can be use in both live observation as well as VR of clinical encounters to identify and promote behaviors associated with patient-centered care, most of which also overlap with behaviors associated with Emotional Intelligence (EI). We propose a VR that is structured less on a seek-and-find of clinician behaviors and more as a self-reflective exercise of how the clinician presents in the room alongside how they were feeling during that encounter. We believe that this exercise promotes the first two skills of EI (self-awareness and self-management) and then provides a foundation on which to build the second pair of skills (social awareness and relationship management). This perspective paper offers guidance, including stepwise instruction, on how to facilitate such a VR curriculum.

## Introduction

Live and/or video observation of the clinical encounter is a longstanding training practice in medical residencies and other training programs for health care professionals. Depending on the available resources, clinicians in training may practice in patient rooms enabled with one-way observation mirrors [a practice more frequently utilized within the behavioral health field (Sehgal et al., 2014)], have a supervisor accompany them into the patient room to observe selected clinical encounters, or video or audio record themselves during clinical encounters for later review. With the expense and architectural/logistical challenges of one-way mirrors (i.e., designation of adjoining observation rooms) and the intrusiveness for both trainee and patient of having a supervisor in the room during the clinical encounter, Video Review (VR) has been a commonly adopted form of clinical observation in medical schools and residencies (Muench et al., 2013). Notably, VR also offers a “playback” feature for the learner, who is able to retrospectively view and reflect on their patient interaction.

Despite the continued utilization of VR as a training method, there is some debate around the application and role of VR and the logistics of operationalizing a VR-program (Muench et al., 2013; Sherman et al., 2018). There are also ethical considerations of using VR, including intrusion upon the patient-clinician relationship, confidentiality, and informed consent (Foster, 2016). The discussion of informed consent for VR is outside the scope of this article but warrants mentioning. Briefly, in order to be practiced ethically, VR must be facilitated in a setting where patients and/or their guardians have been fully apprised of what VR entails, feel able to consent or dissent to VR at any point in the care encounter without this affecting their care, and are aware of how their video will be used, who will view it, and where and how long it will be stored. Similar considerations must be made for observations of clinical encounters in which a recording is not made (i.e., live in-the-room supervision or observation through a one-way mirror) with the distinction that outcome of a recording requires additional consideration around storage of protected health information containing data and who will have access in the future.

At least a dozen VR/observation templates and rating scales have been developed to support supervisors’ review of patient encounters. These forms, which are typically connected to larger curricular programs that provide training in the art of facilitating patient-centered care, include Common Ground Instrument (Lang et al., 2004), Patient Centered Observation Form (PCOF) (Keen et al., 2015), and the four habits coding scheme (Krupat et al., 2006). With a focus on the presence or absence of clinician behaviors (i.e., eye contact, use of open-ended questions, balance of technology use with patient interaction), suggesting that for most training programs, the purpose of the VR is to confirm whether or not clinicians in training possess a number of behaviors consistent with the behaviors of a “good clinician”.

A recent Delphi study by Zulman with senior author, surgeon and novelist, Abraham Verghese, found the following to be associated with physician presence and patient connection: (1) preparing with intention; (2) listening intently and completely; (3) agreeing on what matters most; (4) connecting with the patient’s story; and (5) exploring emotional cues (notice, name, and validate the patient’s emotions) (2020). These practices are foundational and grounded in the clinician’s emotional intelligence (EI). In their book and program, *emotional intelligence 2.0*, Bradberry and Greaves (2009) break EI into two pairs of skills (four skills total): self-awareness and self-management (personal competence skills) and social awareness and relational management (social competence skills). According to the authors, these skills build on one another such that as individuals (in both personal and professional capacities) increase their personal competence, they lay the foundation for social competence. While the Accreditation Council for Graduate Medical Education (ACGME, 2022) does not operationalize EI in their milestones for family medicine physicians, expectations for “Professionalism” and “Interpersonal and Communication Skills,” are consistent with Bradberry and Greaves’ skills. Historically, VR practices have focused on observable behaviors that convey social awareness and relational management EI skills [i.e., eye contact, balancing technology use with warm introduction and greeting, as included on the PCOF (Keen et al., 2015)]; the training we have developed for our residency program incorporates the self-awareness and self-management skills of EI. We propose that EI is a framework from which to conceptualize family medicine residents’ ability to emotionally connect with patients.

For example, a resident who has achieved a Level 3 (mid-level) competence in the ACGME milestones in accountability/conscientiousness (within the category of Professionalism), “Performs tasks and responsibilities in a timely manner with appropriate attention to detail in complex or stressful situations (and) proactively implements strategies to ensure that the needs of patients, teams, and systems are met” (p. 15). On the milestone of “Patient- and Family-Centered Communication” (within the category of Interpersonal and Communication Skills), a resident who has achieved a Level 3 competence, “Establishes a therapeutic relationship in challenging patient encounters (and) When prompted, reflects on personal biases while attempting to minimize communication barriers (and) sensitively and compassionately delivers medical information, managing patient/family values, goals, preferences, uncertainty, and conflict” (p. 17).

We assert a VR that is structured less on a seek-and-find of clinician behaviors that are associated with EI and more as a self-reflective exercise of how the clinician presents in the room alongside how they were feeling during that presentation (e.g., formal vs. informal, boundaried vs. interpersonally connected, energetic vs. calm). We believe that this exercise promotes the first two skills of EI (self-awareness and self-management) and then provides a foundation on which to build the second two (social awareness and relationship management) through turning attention to how the patient is reacting to and receiving the clinician’s care.

As of this writing, there have been recent changes to the ACGME family medicine program requirements that deprioritize direct observation of resident as a means of performance evaluation and method for “improvement toward unsupervised practice” (p.48). It is our sincere hope that residencies will continue to invest in the technologies that allow for direct observation, and may find utility in this suggested curriculum to structure this resident learning experience.

## Recommended application

Over the course of their residency, a more behaviorally-based VR (using the Common Ground Instrument and/or PCOF depending on facilitator preference) is conducted during first-year and second-year orientation. The reflection-based EI-building VR process is completed twice [once with first-year residents on Behavioral Health Rotation-1 (BHR-1) and once with second-year residents during Behavioral Health Rotation-2 (BHR-2)], for a total of four VRs, two of which follow the recommended application presented here, during residency (see Table 1 for timing of VR curricula during three-year family medicine residency).

**Table 1:** Timing of VR curricula during 3-year family medicine residency.

Timing during fam med residency	VR curricula type
Year 1 orientation	Behaviorally-based VR
Year 1 BH rotation (BHR-1)	Reflection-based EI-building VR
Year 2 orientation	Behaviorally-based VR
Year 2 BH rotation (BHR-1)	Reflection-based EI-building VR

The setting for the VR is a small conference room with two to four family medicine residents and two facilitators, including a medical faculty member and a behavioral science faculty member. At the beginning of their two-week Behavioral Health Rotation, residents are asked to record at least one care encounter in the teaching clinic to review at the end of the rotation with their co-residents also on rotation, as well as the two facilitators (see Table 2 for lesson plan for EI-based VR curriculum). In this way, residents have control over which care encounter is selected for viewing. They are encouraged to record multiple encounters so they have a choice and are reassured that the chief complaint need not be behavioral – any encounter in which the patient consents to recording is viable for the VR. Each individual residents’ VR is given approximately 30 min.

**Table 2:** Lesson plan for EI-building VR curriculum.

*Two weeks prior to VR*
Resident asked to record at least one medical encounter in clinic.
Patient consent for video recording is obtained. Video is recorded and made available to facilitators on secure network.
*Day of VR*
Three spectra exercise Facilitators set ground rules Individual VR Brief background Resident is asked “Is there any particular feedback you would like?” Video is paused periodically, or at request, to reflect on resident presentation/experience, patient presentation, and resident adjustments based on patient presentation. Both residents and peers are encouraged to make these reflections. Questions to promote self-awareness/management may include: “So far, has your presentation in this encounter matched up with what you stated was your general presentation in the room? Why or why not?” Questions to promote social awareness/relational management may include: “What did you notice about the patient’s presentation that communicated to you the need for adjustment in your presentation? How did it go? Did you have to make further adjustments or course corrections?” Observations and feedback are strengths-based and curious. Resident is asked at the end of their video, “What did you notice yourself doing well?” and “Is there anything you would do differently next time?” Peers are asked, “What would like to borrow from your co-resident’s practice?”
Repeat with adequate breaks during process.
Debrief: “How was it to watch yourselves, each other, and be watched by each other? What did you learn/note?”

**Figure 1:**
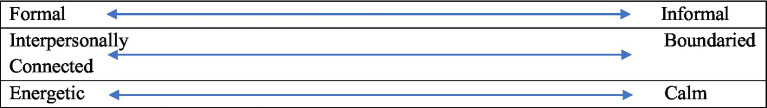
Three spectra exercise.

Our initial approach to implementing a reflection-based EI-building VR curriculum was to structure the review around self-awareness and then peer and facilitator observations of residents’ presentation in the room. We ask residents to rate themselves (subjectively) on the following spectra: formal to informal, emotionally interconnected to boundaried, and energetic to calm (see Figure 1). The prompt for this rating exercise is: “Given you likely adjust your presentation based on a myriad of factors (how you are feeling, how the patient is doing, and how well you know them, etc.), how would you say you *generally* present in the exam room for a medical encounter with a patient?”.

Care was taken to select words with a neutral opposed to positive or negative valence. For example, “formal vs. informal” was used instead of “professional vs. unprofessional” and “interpersonally connected vs. boundaried” instead of “warm vs. cold.” Facilitators also make a point to avoid stacking terms often associated with one another all on the same side of the spectra (i.e., informal, interpersonally connected, and energetic are distributed across both sides of the spectra rather than together on one side).

Either by taking turns marking their initials on each of the three spectra, or having the facilitator approximate the residents’ verbal report (e.g., “So about halfway between formal and informal?” or “About a quarter of the way out from informal?”), the residents’ rating of their general presentation is gathered. Notably, facilitators also engage in this exercise, rating themselves on how they typically present in the exam room. Typically, facilitators complete their own ratings first, by way of modeling, and/or give examples of how clinicians may present in the room in order to ground residents in the meaning of the spectra terms. Once completed, residents are told that this discussion will be revisited during each of their VRs.

Before beginning, ground rules are set around the VR. The facilitators state that the assumption is made that the medical care in these VRs is above reproach; the focus instead is on resident-patient interactions and encounter management. Further, the facilitators share that this is a strength-based approach where the aim is to expand what is going well, rather than to hone in on what is going poorly. Next each resident takes turns having their video reviewed (in no particular order). Before playing the video, the resident is asked to give a brief background on the patient (e.g., chief complaint, whether this is a new or continuity patient). The resident is also asked if there is any particular feedback that they would like from peers and facilitators (e.g., residents might ask, “Could you let me know how I balance use of technology with my rapport-building efforts?” or “Do I give the patient enough time to tell their story and/or appropriately redirect them when time is running short?”).

Over the next half hour, the facilitator will either periodically pause the video to allow for discussion or pause the video when the resident whose video is being reviewed or other residents/facilitator request this. During these pauses, the resident is asked what they notice going on in the video, what they observe in their own behavior, and how they were feeling and/or making a determination to behave as they did at that point in the encounter (self-awareness). Attention is also drawn back to the spectra with questions like, “So far, has your presentation in this encounter matched up with what you stated was your general presentation in the room? Why or why not?” This prompts the resident to begin thinking about how they employed self-management in the encounter.

Perspective taking is also encouraged by asking the other residents what they notice about their peers’ presentation, whether or not this matches with the self-ratings, and why they think their peer may have adjusted their general presentation in this particular encounter (awareness of other’s self-management). In asking circular questions of residents about their peers’ clinical presentations, social awareness is fostered first in encouraging them to relate to their peers (and what they would have done, had they found themselves in the same situation), and then in thinking about the patients’ experience in the room, as well.

Through this line of questioning and discussion, the focus is shifted towards social competence skills: social awareness and relational management. The facilitator might ask the following questions of the resident: “What did you notice about the patient’s presentation that communicated to you the need for adjustment in your presentation? How did it go? Did you have to make further adjustments or course corrections?” This highlights the utility of the clinician to adjusting their own presentation to either match, or in some cases balance, the presentation of the patient. For example, the facilitator could share that when a patient comes into the clinic brimming with nervous energy, a calm presence paired with words that validate and normalize the patient’s concerns is often effective in dialing down the patient’s anxiety.

Facilitators are charged with maintaining emotional safety throughout the VR. Affirmations and compliments are given freely in an effort to expand solutions and successes. On the other hand, concerns are approached as curiosities with awareness that residents are especially vulnerable in this exercise, on the stage as it were, in front of two facilitators and up to three of their peers. Any major concerns should be addressed one-on-one at another time.

When the video is over (note, if the video is longer than 30 min, the facilitator will need to negotiate with the resident which parts of the video will be viewed vs. skipped over with the objective of getting a sampling of the opening, middle, and closing parts of the encounter), facilitators guide a strengths-based discussion by asking resident and their peers to reflect on the video as a whole. They may ask of the resident, “What did you notice yourself doing well?” and “Is there anything you would do differently next time?” Facilitators may ask of the resident’s peers, “What would like to borrow from your co-resident’s practice?”.

When all residents’ videos are reviewed (with adequate breaks throughout the process), facilitators take 10–15 min to debrief on the process. How was it to watch themselves, each other, and be watched by each other? What did they learn/note? We have observed that residents will often comment here that that the process was “not as bad” as they anticipated when it came to watching themselves/being observed, and that they appreciated the opportunity to see their peers in action.

## Conclusion

Video review is a tried-and-true teaching method for healthcare professionals. In particular, it is employed to aid in rapport-building, agenda-setting, and encounter management practices. We propose an emotionally-intelligence based VR curriculum that replaces a behavioral focus with attention to residents’ self-reflection of their internal experience in the room (self-awareness), the externalization of that experience (self-management), the perception of the patient in the room (social awareness), and their ability to integrate their own and patient perceptions, behaviors, and agendas/needs (relational management). In a rapidly changing landscape of healthcare technologies that attempt to improve access, but sometimes at the expense of space and time for patient-clinician interaction, the use of video review technology can help us get back to the basics of family medicine: continuity and connection.

## Data availability statement

The original contributions presented in the study are included in the article/Supplementary material, further inquiries can be directed to the corresponding author.

## Author contributions

AK and MK conceptualized, developed, and executed the curriculum with family medicine residents. AK wrote the first draft of the manuscript with MK reading and improving subsequent drafts. PB, LN, EC-Q, and SD contributed support and refined interpretation for the EI framework as applied to residency education, with backgrounds in developmental psychology (PB and SD) and clinical psychology in medical residency settings (LN and EC-Q). All authors contributed to the article and approved the submitted version.

## Conflict of interest

The authors declare that the research was conducted in the absence of any commercial or financial relationships that could be construed as a potential conflict of interest.

## Publisher’s note

All claims expressed in this article are solely those of the authors and do not necessarily represent those of their affiliated organizations, or those of the publisher, the editors and the reviewers. Any product that may be evaluated in this article, or claim that may be made by its manufacturer, is not guaranteed or endorsed by the publisher.
